# Epidemiology of COVID-19 cases and vaccination coverage in Seremban District, Malaysia, 2021

**DOI:** 10.5365/wpsar.2023.14.2.985

**Published:** 2023-05-24

**Authors:** Khairul Hafidz Alkhair Khairul Amin, Nur Nadiatul Asyikin Bujang, Siti Aishah Abas, Nadiatul Ima Zulkifli, Syuaib Aiman Amir, Sharina Mohd Shah, Veshny Ganesan, Nurul Fazilah Aziz, Muhammad Adli Jalaluddin, Mohd Shahrol Abd Wahil, Muhamad Hazizi Muhamad Hasani, Noor Khalili Mohd Ali, Mohamad Paid Yusof

**Affiliations:** a Seremban District Health Office, Ministry of Health Malaysia, Seremban, Negeri Sembilan, Malaysia.; b Department of Community Health, Faculty of Medicine and Health Sciences, Universiti Putra Malaysia, Serdang, Selangor, Malaysia.; c Department of Social and Preventive Medicine, Faculty of Medicine, Universiti Malaya, Kuala Lumpur, Malaysia.; d Department of Public Health Medicine, Faculty of Medicine, Universiti Teknologi MARA, Shah Alam, Selangor, Malaysia.; e Disease Control Division, Ministry of Health, Putrajaya, Malaysia.

## Abstract

**Objective:**

Malaysia’s first case of coronavirus disease (COVID-19) was reported in January 2020, with the first case in the state of Negeri Sembilan diagnosed on 17 February 2020. The National COVID-19 Immunization Programme commenced in early March 2021 in Negeri Sembilan. This study describes the COVID-19 cases and vaccination coverage in Seremban District, Negeri Sembilan, during 2021.

**Methods:**

The demographic and clinical characteristics of COVID-19 cases and the district’s vaccination coverage were described. Vaccination coverage was plotted against COVID-19 cases on the epidemic curve. The χ^2^ test was used to examine the differences between the vaccination status of COVID-19 cases and severity category, hospitalization status and mortality.

**Results:**

In Seremban District, there were 65 879 confirmed cases of COVID-19 in 2021. The data revealed that the  21–30-year age group had the highest proportion of cases (16 365; 24.8%), the majority of cases were male (58.3%), and most cases were from the subdistrict of Ampangan (23.1%). The majority of cases were Malaysian. Over half (53.5%) were symptomatic, with fever (29.8%) and cough (22.8%) being the most frequently reported symptoms. COVID-19 vaccination status was significantly associated with severity category, hospitalization and mortality (*P* < 0.001 for all categories).

**Discussion:**

This is the first study to describe two-dose vaccination coverage and the trend in COVID-19 cases in Seremban District. It was observed that COVID-19 cases had been reduced following more than 60.0% vaccination coverage.

In Malaysia, the first case of coronavirus disease (COVID-19) was diagnosed on 25 January 2020. In the urban city of Seremban, which is the state capital of Negeri Sembilan with a population of 636 400, the first case was diagnosed on 5 February 2020. (*1*) Malaysia initiated the National COVID-19 Immunization Programme on 24 February 2021, which commenced in Negeri Sembilan on 3 March 2021. (*2*) The programme provided free COVID-19 vaccines across three phases: Phase 1 targeted front-line health-care workers; Phase 2 commenced on 19 April 2021 for elderly adults and high-risk groups; and Phase 3 began on 12 July 2021 for all eligible people over the age of 18.

Herd immunity for COVID-19 was estimated to require 50–66% of the population to be immunized, either spontaneously or artificially, (*3*) and the Ministry of Health Malaysia projected a herd immunity threshold of 70–80% vaccination coverage. (*4*) To the best of our knowledge, there has been no local study on COVID-19 vaccination in Negeri Sembilan; therefore, the objective of this study is to describe the characteristics of COVID-19 cases and two-dose vaccination coverage in Seremban District during 2021.

## Methods

A descriptive analysis of all COVID-19 cases registered in Seremban was undertaken from 1 January to 31 December 2021. A confirmed case of COVID-19 was defined as a person with a positive rapid antigen test in predetermined areas with an incidence of COVID-19 greater than 10% OR a person (alive or dead) with a positive reverse transcription polymerase chain reaction test. (*5*) COVID-19 severity was classified into five categories: category 1, asymptomatic; category 2, symptomatic without pneumonia symptoms; category 3, symptomatic with pneumonia symptoms; category 4, requiring intensive care and supplemental oxygen; and category 5, critical illness with multiple organ involvement. (*6*)

**Fig. 1:**
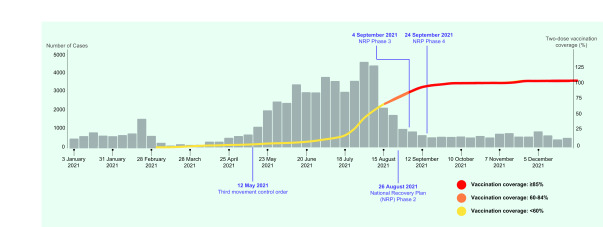
Number of COVID-19 cases by week and vaccination coverage in Seremban District, Malaysia, 1 January to 31 December 2021

Telephone interviews for every case were conducted by employees of the Seremban District Health Office to gather data on demographics, symptoms, onset date, date of exposure, travel history, comorbidities and vaccination status. Vaccination coverage for Seremban District from March to July 2021 was obtained from data compiled manually in Microsoft Excel® from each health-care facility and the Malaysia Vaccine Administration System. From 23 July to 31 December 2021, vaccination coverage was obtained through an automated system. (*7*) Vaccination coverage was plotted against COVID-19 cases on an epidemic curve (**Fig. 1**).

All verified data were recorded in a line list, and Microsoft Excel® was used for data analysis. The demographic and clinical characteristics of confirmed COVID-19 cases and district vaccination coverage were tabulated and analysed using descriptive statistics. The χ^2^ test was used to examine the differences between the vaccination status of COVID-19 cases and severity category, hospitalization status and mortality.

## Results

There were 65 879 confirmed cases of COVID-19 in Seremban District in 2021, giving an incidence rate of 10 358 per 100 000 population. The cases were distributed unevenly among the eight subdistricts. Subdistrict Ampangan recorded the highest number of cases  (15 213; 23.1%), while subdistrict Pantai had the lowest (362; 0.5%). A plurality of cases were aged 21–30 years (16 365; 24.8%), and a majority were male (38 421; 58.3%), Malaysian nationals (54 023; 82.0%) and symptomatic (35 262; 53.5%). Fever (19 602; 29.8%), cough (15 049; 22.8%) and loss of smell and taste (5448; 8.3%) were the most frequently observed symptoms. The majority of cases had no comorbidities (55 981; 85.0%) and had a history of close contact with at least one other confirmed case (47 480; 72.1%). Almost all of the reported cases (65 642; 99.6%) were locally acquired, 23 333 (35.4%) were hospitalized for isolation and treatment, and 561 died (0.9%) (**Table 1**).

**Table 1:** Characteristics of COVID-19 cases in Seremban District, Malaysia, 1 January to 31 December 2021 (*n* = 65 879)

Characteristic	*n*	%
**Age group**		
0–10	9075	13.8
11–20	8236	12.5
21–30	16 365	24.8
31–40	12 576	19.1
41–50	7190	10.9
51–60	4965	7.5
> 60	3947	6.0
No information	3525	5.4
**Sex**		
Male	38 421	58.3
Female	27 458	41.7
**Nationality**		
Malaysian	54 023	82.0
Other	11 856	18.0
**Symptomatic**		
Yes	35 262	53.5
No	30 617	46.5
**Subdistrict**		
Ampangan	15 213	23.1
Labu	13 445	20.4
Setul	10 761	16.3
Rantau	9286	14.1
Rasah	7278	11.0
Seremban	6558	10.0
Lenggeng	2038	3.1
Bandar Seremban	938	1.4
Pantai	362	0.5
**Symptoms**		
Fever	19 602	29.8
Cough	15 049	22.8
Loss of smell and taste	5448	8.3
Sore throat	3572	5.4
Myalgia	2760	4.2
Headache	2096	3.2
Stomach pain	1155	1.8
**Comorbidities**		
None	55 981	85.0
Hypertension	5508	8.4
Diabetes mellitus	3731	5.7
Asthma	1408	2.1
Heart disease	652	1.0
Dyslipidaemia	477	0.7
**History of close contact with confirmed COVID-19 case**		
Yes	47 480	72.1
No	18 399	27.9
**Source of infection**		
Local	65 642	99.6
Imported	237	0.4
**Hospitalized**		
Yes	23 333	35.4
No	42 546	64.6
**Status**		
Alive	65 318	99.1
Dead	561	0.9

The number of COVID-19 cases per week increased between March and August 2021, declined in early August 2021, and then plateaued until December 2021. On 8 August 2021, two-dose vaccination coverage for adults reached 56% (**Fig. 1**).

[**insert** Figure 1]

Before the vaccination programme, from 1 January to 20 March 2021, there were 7149 confirmed COVID-19 cases including 31 deaths. Most of these cases were in severity categories 1 (4807; 67.2%) and 2 (2297; 32.2%), while 14 cases (0.2%) were in category 3. None were in categories 4 and 5 (**Table 2**).

From the start of the vaccination programme on 21 March 2021 until 60.0% coverage was reached on 15 August 2021, 43 375 patients were registered with COVID-19, of whom 37 937 (87.5%) were unvaccinated. Of the 476 deaths, 431 (90.5%) were unvaccinated. In terms of severity, 23 265 were category 1 (21 316 unvaccinated vs 1949 vaccinated), 18 970 were category 2 (15 679 unvaccinated vs 3291 vaccinated), 656 were category 3 (507 unvaccinated vs 149 vaccinated), 7 were category 4 (4 unvaccinated vs 3 vaccinated), and 1 was category 5 (vaccinated) (**Table 2**).

**Table 2:** COVID-19 cases by severity category before and after the vaccination programme started in Seremban District, Malaysia, 1 January to 31 December 2021 (*n* = 65 879)

Before vaccination programme, 1 January to 20 March 2021					
Severity category		Cases (*n* = 7 149)			
		*n*		%	
1		4807		67.2	
2		2297		32.2	
3		14		0.2	
4		0		0	
5		0		0	
Deaths		31		0.4	
**Vaccination coverage < 60.0%, 21 March to 14 August 2021**					
**Severity category**	**Cases** **(*n* = 43 375)**	**Unvaccinated** **(*n* = 37 937)**		**Vaccinated** **(*n* = 5 438)**	
		***n***	**%**	***n***	**%**
1	23 265	21 316	91.6	1949	8.4
2	18 970	15 679	82.7	3291	17.3
3	656	507	77.3	149	22.7
4	7	4	57.1	3	42.9
5	1	0	0	1	100
Deaths	476	431	90.5	45	9.5
**Vaccination coverage 60.0–84.0%, 15 August to 5 September 2021**					
**Severity category**	**Cases** **(*n* = 4 965)**	**Unvaccinated** **(*n* = 2 140)**		**Vaccinated** **(*n* = 2 825)**	
		**n**	**%**	**n**	**%**
1	3048	1389	45.6	1659	54.4
2	1771	693	39.1	1078	60.9
3	82	31	37.8	51	62.2
4	12	5	41.7	7	58.3
5	3	2	66.7	1	33.3
Deaths	49	20	40.8	29	59.2
**Vaccination coverage > 85.0%, 6 September to 31 December 2021**					
**Severity category**	**Cases** **(*n* = 10 390)**	**Unvaccinated** **(*n* = 2 353)**		**Vaccinated** **(*n* = 8 037)**	
		***n***	**%**	***n***	**%**
1	4976	1450	29.1	3526	70.9
2	5244	886	16.9	4358	83.1
3	144	12	8.3	132	91.7
4	18	3	16.7	15	83.3
5	3	2	66.7	1	33.3
Deaths	5	0	0	5	100

Category 1: asymptomatic.

Category 2: symptomatic without pneumonia symptoms.

Category 3: symptomatic with pneumonia symptoms.

Category 4: requiring intensive care and supplemental oxygen.

Category 5: critical illness with multiple organ involvement.

For the period of 15 August to 5 September 2021 (with vaccination coverage of 60.0–84.0%), 4965 COVID-19 cases were reported. With regard to COVID-19 severity, 3048 cases were category 1 (1389 unvaccinated vs 1659 vaccinated), 1771 were category 2 (693 unvaccinated vs 1078 vaccinated), 82 were category 3 (31 unvaccinated vs 51 vaccinated), 12 were category 4 (5 unvaccinated vs 7 vaccinated), and 3 were category 5 (2 unvaccinated vs 1 vaccinated) (**Table 2**). Twenty of the 49 COVID-19 deaths (40.8%) during this period were unvaccinated. There was a large decline in cases once vaccination coverage of more than 60.0% was reached (**Fig. 1**). It was also found that the case fatality rate was higher when vaccine coverage was less than 60.0% (1.1%) compared to when it was 60.0–84.0% (0.3%).

There was a significant difference in the distribution of unvaccinated and vaccinated (two doses) cases by severity category, hospitalization and mortality (*P* < 0.001; **Table 3**). The proportion of cases being hospitalized or dying who received two vaccine doses was lower compared to those who were unvaccinated (**Table 3**).

**Table 3:** Factors associated with vaccination status in COVID-19 cases in Seremban District, Malaysia, 1 January to 31 December 2021 (*n* = 65 874)^a^

Variable	Unvaccinated (*n* = 49 579)		Vaccinated (*n* = 16 295)		*P*
	*n*	%	*n*	%	
**Severity category**					
1	29 109	58.7	7155	43.9	< 0.001
2	19 819	40.0	8763	53.8	
3	633	1.3	345	2.1	
4	12	0.02	26	0.2	
5	6	0.01	6	0.04	
**Hospitalized**					
Yes	20 075	40.5	3258	20.0	< 0.001
No	29 504	59.5	13 037	80.0	
**Outcome**					
Alive	49 097	99.0	16 216	99.5	< 0.001
Dead	482	1.0	79	0.5	

^a^ Five of the total 65 879 COVID-19 cases are excluded for lack of information on vaccination status.

Category 1: asymptomatic.

Category 2: symptomatic without pneumonia symptoms.

Category 3: symptomatic with pneumonia symptoms.

Category 4: requiring intensive care and supplemental oxygen.

Category 5: critical illness with multiple organ involvement.

## Discussion

This study describes the demographic and clinical characteristics of 65 879 cases of COVID-19 from the most densely populated district in the state of Negeri Sembilan. It demonstrated that the number of cases per week declined after the district vaccination coverage reached 60.0%.

The 21–30-year age group had the highest proportion of COVID-19 cases, possibly due to rapid housing development and a growing workforce in this district. (*8*) The fact that there were more cases among the male population could be due to their being less compliant with preventive measures such as frequent hand washing, face-mask use and stay-at-home orders. (*9*) The high proportion of cases registered among Malaysian nationals is most likely due to international travel restrictions. The high urbanization and population density in Ampangan subdistrict (*10*) may also account for the elevated number of cases. Most COVID-19 cases were asymptomatic and detected through contact tracing. The high proportion of young cases may have contributed to the increased number of asymptomatic individuals, as younger individuals tend to have mild or no symptoms. (*11*) Compared to vaccinated cases, unvaccinated cases had higher proportions of cases in the higher severity categories, hospitalizations and deaths, similar to a previous study from Malaysia, which reported that vaccination could prevent severe COVID-19 illness, hospitalization, intensive care unit admission and death. (*12*)

Our data showed that the number of COVID-19 cases per week was decreasing when two-dose vaccination coverage reached 60.0%. While vaccination has been shown to reduce COVID-19 outbreaks, (*13*, *14*) the impact of other response components also needs to be considered. Malaysia was under its third movement control order from 12 May 2021 to 1 April 2022, during which international, inter-state and inter-district travel, as well as economic, social, educational, sports and business operation hours, were restricted. Physical distancing and mask use were enforced nationwide under the Prevention and Control of Infectious Diseases Act 1988. Personal hygiene practices including hand washing were continuously promoted by the Ministry of Health through various media platforms. During this period, COVID-19 variants Alpha and Beta were mostly circulating in Malaysia before the Delta variant emerged in July 2021. (*15*)

Another intervention for COVID-19 was the establishment of the Greater Klang Valley Special Task Force on 12 July 2021. This task force was a multiagency collaboration for COVID-19 management in the Klang Valley (covering the federal territories of Kuala Lumpur and Putrajaya and the state of Selangor) and Seremban District. The task force’s objectives included organizing strategic actions to improve health-care delivery, lessening the transmission of infectious diseases, and assisting both the general public and health-care professionals. (*16*)

To our knowledge, this is the first study to describe two-dose vaccination coverage and the trend of COVID-19 cases in Seremban District. It was observed that COVID-19 cases decreased once 60.0% vaccination coverage had been reached. The strength of this study is in the use of large data sets acquired from the Seremban District Health Office, which may reflect the real number of COVID-19 cases in other districts. These data are managed systematically, making their source more reliable.

This study has limitations, the first of which is that it is a descriptive observational study of one area in Malaysia. A more sophisticated statistical analysis is needed to compare vaccination coverage and the number of COVID-19 cases. Given that only symptomatic patients were screened for COVID-19, (*15*) a potentially large number of individuals with asymptomatic infection may have remained undiagnosed, thus contributing to the lower number of reported COVID-19 cases. Other limitations include: the lack of data on disease progression and on the use of the severity categories during diagnosis; the unavailability of COVID-19 vaccine for the different variants; and the fact that case data on COVID-19 variants were not obtained during field investigations as they were not a priority for the primary management of COVID-19. The findings of this study need to be interpreted with caution.

In summary, this study describes the epidemiology of COVID-19 cases in 2021 in Seremban District, Malaysia. Although we show that the COVID-19 case numbers decreased as vaccination coverage increased, other control measures such as movement control orders, physical distancing, mask use and regular hand washing are likely to have also contributed to the decrease in cases. Additional analyses are needed to confirm an association between COVID-19 cases and vaccination coverage.
